# Breaking Bad News: A Randomized Trial Assessing Resident Performance After Novel Video Instruction

**DOI:** 10.7759/cureus.15461

**Published:** 2021-06-05

**Authors:** Anthony Shanks, Maria Brann, Jennifer Bute, Vyvian Borse, Tiffany Tonismae, Nikki Scott

**Affiliations:** 1 Obstetrics and Gynecology, Indiana University School of Medicine, Indianapolis, USA; 2 Communications, Indiana University - Purdue University in Indianapolis, Indianapolis, USA; 3 Obstetrics and Gynecology, Johns Hopkins All Children's Hospital, St. Petersburg, USA

**Keywords:** breaking bad news, patient simulation, resident, education, video

## Abstract

Introduction

Delivering bad news to patients is an essential skill for physicians, which is often developed through patient encounters. Residents in our program participate in objective structured clinical examinations (OSCEs) on an annual basis to evaluate their skills in these scenarios. Our objectives were to develop an educational video and determine if an educational video provided to residents prior to OSCEs would improve performance.

Methods

Previous OSCEs were reviewed to identify best practices and to create a four-minute video highlighting the “do’s and don’ts” of delivering bad news. Residents in two post-graduate year (PGY) classes were randomized to watch the video prior to or after a standardized patient encounter. Three masked reviewers assessed resident empathy, attention, and understanding on 10 five-point Likert scales and assigned a total score (scale: 0-50). Hedges’ g was used to assess mean scores and effect size.

Results

A total of 17 residents participated in the evaluation: nine in the pre-OSCE video group and eight in the control group. Residents randomized to the video prior to the patient encounter had a mean score of 37.01 (SD=3.6). Residents randomized to the control group had a mean score of 35.38 (SD=4.85). Hedges’ g was 0.37 (95% CI: -0.59 to 1.33).

Conclusion

Residents randomized to the video group had a small increase in OSCE performance, which was not statistically significant. The novel video was helpful and addresses the need for a quick pre-assessment educational tool, though interns and graduating medical students may be a more appropriate target audience for instruction.

## Introduction

Breaking bad news to patients is an essential skill for physicians. Without proper training, residents may develop inappropriate ways of delivering this information and may be ill-prepared to cope with the emotional repercussions [1]. The discomfort associated with this task may lead physicians to disengage from patients [2], and poor communication of bad news may lead patients to seek a new physician for their care [3].

Learning professionalism begins in medical school [4], but the ability to deliver sensitive information compassionately and effectively is often learned through patient encounters in residency. To augment this training, workshops and simulations have been developed to teach these communication skills [5,6]. Although helpful, these workshops require time and resources to complete, and many study outcomes have focused on self-reflection as opposed to quantifiable improvement in communication skills [1,7-11]. Additionally, many workshops are geared towards medical students and may not necessarily be as relevant for training resident physicians [4,12-14]. In studies utilizing an objective evaluation, the pre-existing resources either provided no pre-assessment educational materials [4,15,16] or provided workshop educational materials that can require as much as eight weeks for completion prior to the assessment [17]. This leaves a gap in the educational material literature for an effective and concise pre-assessment learning tool.

At our residency training program, obstetric and gynecologic (OB/GYN) residents participate in objective structured clinical examinations (OSCEs) to provide a venue for resident evaluation in these scenarios. Residents interact with standardized patients, and the encounters are viewed by faculty to allow feedback to be provided to participants. A prior OSCE study at our institution demonstrated that residents engaged in minimum informed decision-making but did not address all of the communication elements necessary for informed decisions [18]. Additionally, participants in prior OSCE scenarios did not address all standardized patient concerns, and they communicated limited support for the patient’s decision [19].

Today’s learner is comfortable obtaining information in various formats, and the use of a video is an attractive teaching tool [20]. Video has the potential to standardize instruction, provide learners an opportunity to prepare before clinical encounters, and deliver information effectively and concisely.

Our objective for this study was twofold. First, we sought to create a video of best practices to teach OB/GYN residents how to improve their skill with delivering bad news. Second, we sought to determine if this video provided to residents prior to OSCEs would improve performance on standardized patient assessments. Our hypothesis was that residents who provided information on best practices prior to a standardized patient encounter would score higher on OSCE scenarios that require residents to deliver bad news.

This research was presented at the 2018 Council on Resident Education in Obstetrics and Gynecology (CREOG) and Association of Professors of Gynecology and Obstetrics (APGO) Annual Meeting, February 28 to March 3, 2018, in National Harbor, Maryland.

## Materials and methods

Study design

We utilized a single-institution mixed-methods design of OB/GYN residents that combined qualitative and quantitative analysis. The qualitative aspect utilized direct feedback from our OSCE stations to create a novel video. The quantitative aspect evaluated the implementation of the video into future OSCE performance. This aspect was a randomized controlled trial where residents were randomized to the novel video (intervention) or no-video (control) groups prior to a formal evaluation of a scenario delivering bad news. IRB approval was granted by Indiana University.

Educational video creation

Our university OSCE stations are developed to assess residents’ technical skill and clinical acumen. Each post-graduate year (PGY) is assigned a dedicated day at our Simulation Center to be led through a variety of clinical and technical skills to allow for immediate feedback. One station for each PGY is utilized to assess a resident’s ability to deliver bad news to standardized patients. These OSCE encounters are video-recorded to allow for future playback and review.

Intern OSCEs from the graduating classes of 2015-2018 were identified and reviewed (n = 40) [21]. OSCEs in that time frame consisted of counseling a patient on an unexpected miscarriage. The OSCE video recordings were reviewed by members of the study team (M.B., J.B.) and shown in focus group discussions to 22 women who had experienced receiving similar sensitive diagnoses. The women were tasked with identifying positive (do’s) and negative (don’ts) behaviors, which are depicted in Table 1 [21].

**Table 1:** Focus Group Feedback for Delivering Sensitive Information Specific behaviors the focus group identified as important positive and negative behaviors exhibited by healthcare providers when delivering sensitive information [21].

Do	Don’t
Demonstrate empathy	Use medical jargon and loaded terminology
Allow the patient an opportunity to process	Rush the conversation
Check for understanding by asking explicit and direct questions	
Provide full attention to the patient and her needs	
Recognize the uniqueness of the situation for each patient	

The women identified specific behaviors as important do’s and don’ts of breaking bad news [21], which are in agreement with behaviors identified in previous literature [3,22-25] including the SPIKES (Setting, Perception, Invitation, Knowledge, Empathy, Summarize) protocol [26]. Other important behaviors cited in the literature that were not specified by this group include ensuring patients receive bad news in person [25], allotting enough time to address all patient questions [25], arranging for the proper setting [26], asking for an invitation to share information and determining with the patient how much information is desired [26], and warning the patient bad news is coming prior to sharing [26]. Studies further describe important behaviors when delivering bad news, which include elucidating the patient’s perception of his/her health/condition [26], providing the diagnosis [22,25], implications [22] and treatment options [3,26], being honest [3], planning follow-up [22,26], summarizing [26] or providing written information regarding the diagnosis [22], providing referral or information regarding support services [22], and ensuring patients feel supported through the new diagnosis [22]. While not specified by the women in this study, in the literature, when displaying empathy, physical touch is not always considered helpful by patients, and some find it aversive [22]. Additionally, it is important to note that there are cultural differences as well as differences across age, sex, and educational level in patient preferences for receiving bad news [25].

These identified behaviors [21], along with evidence from previous literature [3,22-26] and reviews of our prior OSCE performances, formed the basis of a novel four-minute video highlighting a patient encounter. In this video, members of the study team (T.S., T.T.) performed scenarios where both positive and negative actions were demonstrated. Summary points were provided after each scenario to reinforce positive behaviors.

Subjects

Participating residents were randomized via random number generator to watching the video before (intervention) or after (control) a standardized patient encounter in which they provided counseling following a miscarriage. Two classes of residents undergoing OSCEs formed our study group.

OSCE evaluation

In our Breaking Bad News OSCE scenario, the standardized patient was in the first trimester, asymptomatic, and presented for a review of her ultrasound that noted a nonviable pregnancy. Female standardized patients were provided by the institution. These encounters occurred in a room designed to mimic an outpatient OB/GYN office with adequate equipment to allow for video and audio of the encounter to be captured. Prerequisite knowledge required by learners for this OSCE scenario included the definition of a missed abortion, the common causes of a missed abortion, the options available for treatment of a missed abortion, and the risk/benefits of the options available for treatment. The Breaking Bad News OSCE was one of 10 OSCE scenarios residents rotated through on one day. The duration of each OSCE scenario was 15 minutes for each resident, and each OSCE scenario was different ranging from assessing technical skills to knowledge. A debrief occurred after all the OSCE scenarios were completed. The staffing requirement for the Breaking Bad News OSCE included one standardized patient and one OB/GYN faculty evaluator. The standardized patient and the resident conducted the OSCE in the simulated clinical exam room while faculty reviewers performed the evaluation in a separate room via the video feed.

Learner assessment

**Figure 1:**
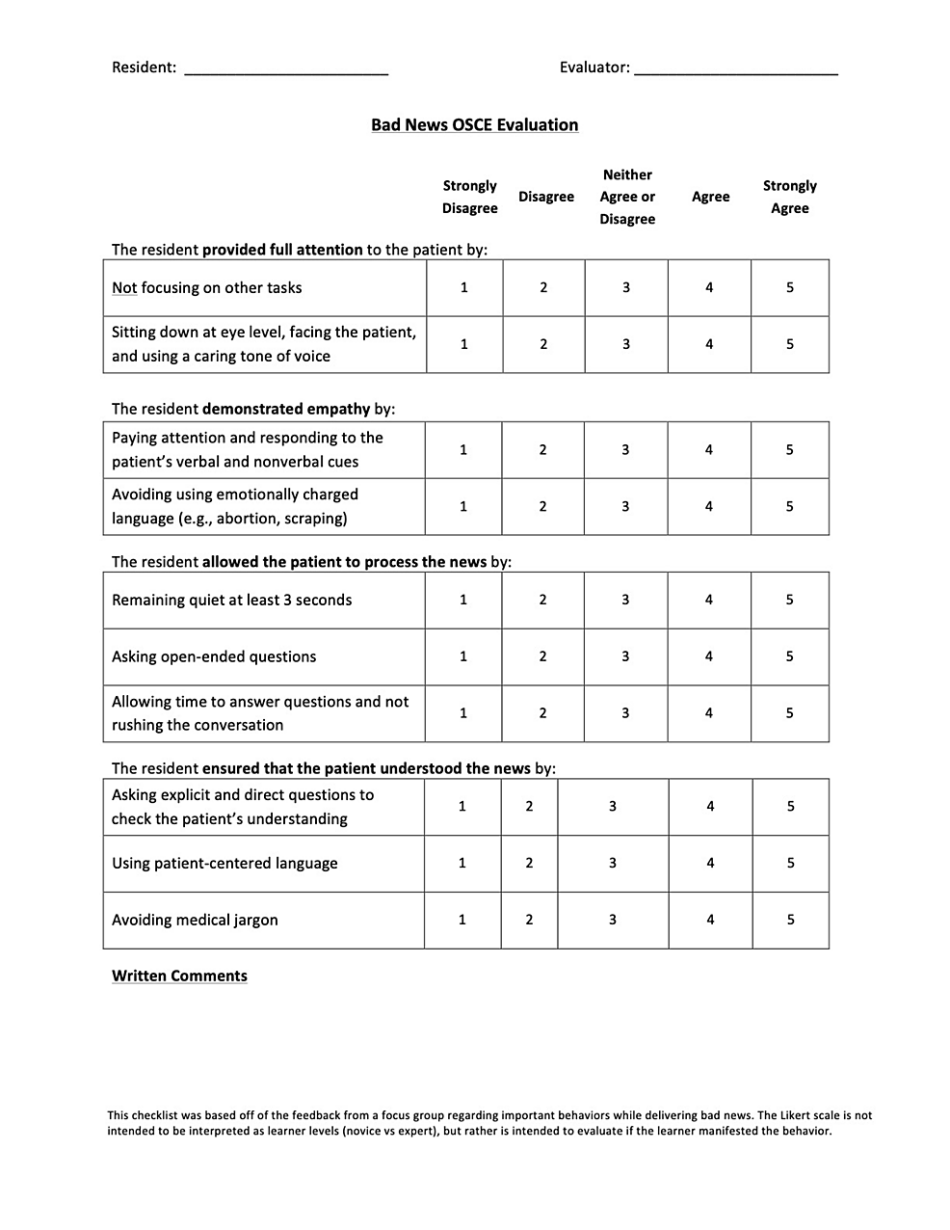
OSCE Evaluation Sheet OSCE, objective structured clinical examinations

There are numerous existing compilations of important behaviors and evaluation tools to assess attributes of professionalism [4,27], and these informed the creation of the evaluation tool utilized in this study. Three reviewers watched the OSCE encounters via a live video feed. Reviewers were blinded and assessed residents on 10 questions that focused on attentiveness, empathy, attention, and understanding. Each of the 10 questions was scored on a Likert scale ranging from strongly disagree (1) to strongly agree (5), and a total score was calculated for each resident (total scale: 5-50). The Likert scale is not intended to score the learner based on level (novice vs. expert), but rather it is intended to evaluate if the learner manifests the behavior. The evaluation was designed around both the do’s and don’ts of Breaking Bad News as illustrated in the video and to reflect other OSCE scenario evaluation tools utilized at our institution. Residents were also asked to self-evaluate their comfort, confidence, stress, and preparation in relation to the scenario in the OSCE (1-5 scale from 1 “strongly disagree” to 5 “strongly agree”) (Figure 1). Mean scores were assessed, and effect size was calculated with Hedges’ g, a measure of effect size used for small sample sizes (<20) [28].

## Results

A total of 17 residents participated in the evaluation from two classes (PGY2 and PGY4). Residents were randomized into two groups: nine in the pre-OSCE video group and eight in the control group. There were more PGY2s assigned to the video group (seven PGY2s, two PGY4s) and more PGY4s assigned to the control group (five PGY4s, three PGY2s). The average time to complete the patient interaction of delivering bad news was 12.24 minutes.

The evaluation focused on Kirkpatrick’s pyramid level 2, Learning, and level 3, Behavior [29]. Learning was assessed through the overall evaluation of the OSCE performance and through the self-reflection afterwards. Behavior was assessed through the overall evaluation of the OSCE performance and through the differences in the overall score between the video and no-video groups.

Residents randomized to the video prior to the patient encounter had a mean score of 37.01 with a standard deviation (SD) of 3.6. Residents randomized to the control group had a mean score of 35.38 with an SD of 4.85. Hedges’ g was 0.37 (95% CI -0.59-1.33). Residents randomized to the control group had a mean self-review of 4.13 (SD=0.23), 4.13 (SD=0.23), 3.13 (SD=0.4), and 4.25 (SD=0.25) for comfort, confidence, stress, and preparedness, respectively. There was no significant difference in self-reported scores with residents randomized to the video who had mean scores of 3.86 (SD=0.26), 3.71 (SD=0.26), 3.14 (SD=0.34), and 3.86 (SD=0.14) for the same categories.

## Discussion

Residents randomized to the video group had a small increase in OSCE performance that was not statistically significant. Residents reported similar levels of comfort, confidence, stress, and preparation, with more than 90% reporting greater than 10 personal clinical encounters of breaking bad news.

The delivery of bad news and difficult information occurs in all medical specialties [5,6]. Appropriate training may assist with physician well-being, help decrease burnout, and improve patient-provider relationships as well as patient satisfaction [1,2]. Additionally, the educational material gap in the literature may well be remedied by a concise educational video.

Our study involved the creation of a novel video to instruct residents on how to deliver bad news followed by an objective assessment of the video’s impact on OSCE performance. This qualitative feedback was instrumental in providing our trainees with feedback from patients in real-life scenarios [21]. The video allowed us to build on previous literature [25,26] and convey best practices in a condensed amount of time in a convenient manner for participants. Our novel video highlighted important behaviors cited in the literature, which include demonstrating empathy [25], providing ample time to allow the patient to process the information [25], and providing full attention to the patient and her needs [26]. Though not specified by our focus group, the literature does describe that when displaying empathy, physical touch is not always considered helpful [22]. Residents rated the video as helpful, though this did not translate into statistically significantly improved OSCE performance in delivering sensitive information.

Limitations of this study include a small sample size, conducting the study at one center, and analyzing results following one set of OSCEs, which all contribute to limiting the generalizability of the findings in this study. An additional limitation is utilizing an assessment tool without behavioral anchors. Future studies could include a larger sample size and multiple centers, utilize multiple sets of OSCEs for analysis, and include residents from multiple specialties (family medicine, emergency medicine, internal medicine, pediatrics, critical care, etc.) and from multiple PGY years. Additional improvements upon this study could include incorporation of behavioral anchors (provide specific behaviors that must be demonstrated to achieve a specific score in each area being evaluated) and updating the educational video to prompt learner thinking and reflection.

Numerous specialties in medicine in addition to OB/GYN require the ability to break bad news to patients empathetically and effectively. This OSCE scenario and preparatory video can be utilized for some specialties including family medicine and emergency medicine among others who may be faced with this situation. Other specialties could utilize the preparatory video and adapt the OSCE scenario to one that is more commonly faced in their field.

Our study used a standardized patient encounter with an objective scoring rubric to examine the benefit of a four-minute video in PGY2 and PGY4 residents. Although participants who viewed the video prior to the OSCE scored similarly to those who were not provided the video, it is important to note that there were more PGY2s in the video group. Though the self-report of delivering bad news was high, it is likely that PGY4s would have more clinical experiences in delivering bad news compared to their PGY2 counterparts; thus, it is possible the video was more beneficial to this group with less experience delivering bad news and helped enable them to perform more like the more experienced residents in delivering bad news. Our sample size is too small to make definite conclusions regarding the impact of video by PGY; however, the video and OSCE assessment may assist those residents who have less clinical experience in delivering bad news. Additionally, interns and graduating medical students may be a more appropriate target audience for future study and intervention.

## Conclusions

Learning to break bad news to patients is an essential skill for physicians. Our study incorporated the available literature and focus group input to develop a concise video with best practices for resident training. Residents randomized to the video group had a small increase in OSCE performance that was not statistically significant. The novel video was helpful and addresses the need for a quick pre-assessment educational tool, though interns and graduating medical students may be a more appropriate target audience for instruction.
